# Neuromuscular Responses of Elite Skaters During Different Roller Figure Skating Jumps

**DOI:** 10.2478/hukin-2014-0029

**Published:** 2014-07-08

**Authors:** Patrícia Dias Pantoja, André Mello, Giane Veiga Liedtke, Ana Carolina Kanitz, Eduardo Lusa Cadore, Stephanie Santana Pinto, Cristine Lima Alberton, Luiz Fernando Martins Kruel

**Affiliations:** 1 School of Physical Education, Federal University of Rio Grande do Sul. Exercise Research Laboratory, Brazil.

**Keywords:** electromyography, elite performance, roller skate jumps

## Abstract

This study aimed to describe the neuromuscular activity of elite athletes who performed various roller figure skating jumps, to determine whether the muscle activation is greater during jumps with more rotations and in which phase the muscles are more active. This study also aimed to analyze if there is any difference in the muscle activity pattern between female and male skaters. Four elite skaters were evaluated, and each participated in two experimental sessions. During the first session, anthropometric data were collected, and the consent forms were signed. For the second session, neuromuscular data were collected during jumps, which were performed with skates at a rink. The following four roller figure skating jumps were evaluated: single Axel, double Axel, double Mapes and triple Mapes. The neuromuscular activity of the following seven muscles was obtained with an electromyograph which was fixed to the waist of each skater with a strap: biceps femoris, lateral gastrocnemius, tibialis anterior, rectus femoris, vastus lateralis, vastus medialis and gluteus maximus. The signal was transmitted wirelessly to a laptop. During the roller figure skating jumps, the lateral gastrocnemius, rectus femoris, vastus lateralis, biceps femoris and gluteus maximus, showed more activation during the jumps with more rotations, and the activation mainly occurred during the propulsion and flight phases. Female skaters demonstrated higher muscle activities in tibialis anterior, vastus lateralis, vastus medialis and gluteus maximus during the landing phase of the triple Mapes, when compared to their male counterparts. The results obtained in this study should be considered when planning training programs with specific exercises that closely resemble the roller figure skating jumps. This may be important for the success of elite skaters in competitions.

## Introduction

Figure skating is a sport that may be performed on ice or on other surfaces. Because the surfaces vary, different skates are used for each of the two types of figure skating (Turner, 1997). The free skating programs of roller figure skating, similarly to those in ice figure skating, require diverse skills, such as jumps, spins, step sequences, spiral sequences and transition movements between these elements (King, 2005; Smith, 2000). Including difficult jumps in competition routines, especially at the international level, usually results in high scores. Therefore, the jumps of elite figure skaters have been studied to provide better knowledge about the strategies used to execute an optimal jump (King et al., 1994; King et al., 2001; King et al., 2002).

Jumps can be performed with one or more rotations, except for the Axel jump, which includes a half rotation (e.g., one and a half rotation for the single Axel and two and a half rotations for the double Axel). According to King (2005), to successfully perform a figure skating jump, skaters must develop the power required in these jumps focusing on exercises that include concentric and eccentric contractions of lower limb extensor muscles. For example, skaters can use light resistance such as dumbbells or sport cords focusing on fast powerful movements. Squats can be performed with dumbbells, emphasizing speed of movement as they explode upward. Box jumps and power skips are also effective techniques to train these muscles using concentric and eccentric contractions at high speeds. Some ice skating studies indicated that the muscles that are important during jumps include the *quadriceps* muscle group, the *hamstrings*, the *gluteus maximus* and the *gastrocnemius* (Aleshinsky et al., 1988; King, 2005; Poe et al., 1994). However, data on the neuromuscular activity patterns that occur while skaters perform figure skating technical elements are difficult to find.

Aleshinsky et al. (1988) suggested that the force production of the *quadriceps femoris* and *hamstrings* muscles differs for the take-off and landing legs of ice skaters. In agreement with this suggestion, Taylor and Psycharakis (2009) found in their study a greater muscle activity for the *rectus femoris* and *biceps femoris* of the take-off leg compared to the landing leg, during the double toe-loop jump. The *gluteus maximus* also appears to have an important role in figure skating jumps. King (2000) reported that the *gluteus maximus* is most likely the main hip extensor used during skating jumps. Thus, specific training of this muscle may be a crucial factor for the realization of high jumps with many rotations (Aleshinsky et al., 1988).

There is little information in the literature regarding neuromuscular activity in ice figure skating. In addition, further studies are necessary to verify the neuromuscular activity data from various muscles during roller figure skating jumps. It is not known if jumps with more rotations require greater muscle activity than jumps with less rotation in elite roller figure skaters and what the muscle activity pattern is during the different phases of these jumps. Taylor and Psycharakis (2009) evaluated one national-level ice skater and found greater activation of the muscles of the take-off leg during jumps with more rotations. This result could be observed in roller figure skaters, however, studies evaluating neuromuscular activity during jumps performed by roller figure skaters are scarce. This information may be valuable for improving a skater’s technical ability. Thus, this study aimed to analyze the neuromuscular responses of elite skaters during different roller figure skating jumps to determine the typical characteristics of the more highly rated jumps, thus expanding knowledge about both the activity of different muscles in high-level athletes and the contribution of these muscles to the performance of roller figure skating jumps. This study also aimed to analyze if there is any difference in the muscle activity pattern between male and female skaters. The hypotheses of the present study were that muscle activity was greater during jumps with more rotations and during the take-off phase as compared to the landing phase, and that muscle activity was different between male and female skaters.

## Material and Methods

### Participants

Four elite roller figure skaters who compete in world championships (three of the four athletes were medalists in a world championship) were invited to participate in the present study, with the consent and collaboration of the respective coaches and the Roller Figure Skating Federation. One female and one male who compete at the Junior level (age: 17 and 19 years; body height: 1.62 and 1.74 m; body mass: 53.2 and 64.2 kg; body fat: 12.4 and 12.2%, respectively) and one female and one male who compete at the Senior level (age: 23 and 25 years; body height: 1.60 and 1.72 m; body mass: 54.4 and 67.7 kg; body fat: 21.9 and 9.1%, respectively). It was not possible to evaluate skaters of different levels, since the analysis included triple jumps that only these elite skaters were able to perform. The skaters were free from any injury that could prevent them from executing the jumps, and the participants reported taking no medications that could affect their performance. Once informed about experimental procedures and risks, all athletes provided written consent for participating in this investigation. The skater that had the age of 17 years provided written parental consent as well. The athletes’ coaches also gave their written consent.

### Experimental Design

The athletes participated in two sessions for measuring anthropometric and neuromuscular data. During the first session, anthropometric data were collected, personal data were recorded, and the consent forms were signed. The second session was dedicated to measuring muscle activity during the skaters’ jumps, at the skating rink.

### Procedures

The protocol was approved by the Research Ethics Committee of the Federal University of Rio Grande do Sul, respecting the *Helsinki Declaration*. The body mass and height measurements were obtained using an analog medical scale and a stadiometer (ASIMED, Barcelona, Spain). Skinfolds were measured using a plicometer (CESCORF, Porto Alegre, Brazil) and to estimate the corporal density we used the Jackson and Pollock (1978) protocol for males and the Jackson et al. (1980) protocol for females. Body fat percentage was estimated using the Siri (1993) equation.

**Figure 1:**
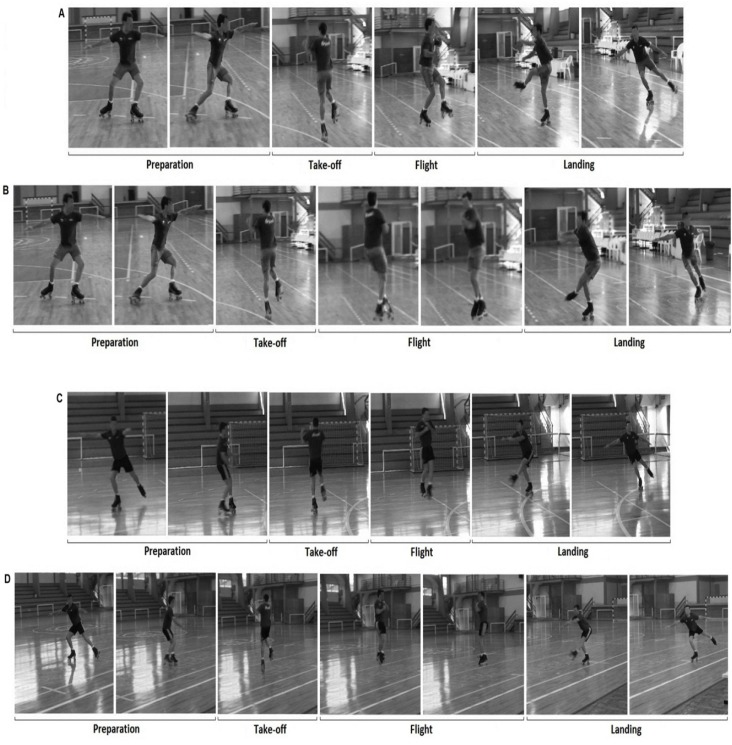
Single Axel (A); double Axel (B); double Mapes (C) and triple Mapes (D) in each phase: preparation, take-off, flight and landing

The neuromuscular activity of the *rectus femoris*, *vastus lateralis*, *vastus medialis*, *gluteus maximus*, *gastrocnemius lateralis*, *tibialis anterior* and the short head of the *biceps femoris* was obtained from the left leg of skaters while they performed the following jumps: single Axel, double Axel, double Mapes and triple Mapes (Figure 1). The Mapes jump in roller figure skating is similar to the toe loop jump, which is performed in ice figure skating. The left leg for both Axel and Mapes jumps is the take-off leg. However, the Axel jump starts when the left leg is in contact with the ground, and the Mapes jump starts while the skater has his or her body weight over the right leg. During the preparation of the Mapes jump the skater transfers his or her body weight from the right leg to the left leg, pressing the toe pick against the floor, which allows the skater to perform the take-off phase from the left leg.

Before the electrodes were positioned, hair was shaved from the electrode placement sites, and the skin in these areas was abraded and cleaned with alcohol; therefore, the inter-electrode resistance, measured with a multimeter (DT-830B, SMART; Glasgow, UK), was low (<3 kΩ) (DeLuca, 1997). Surface monopolar electrodes with 15 mm radii (model mini Medi-Trace 100, Kendall Ag/AgCl; Tyco, USA) were placed in a bipolar configuration over the belly muscle and parallel to the orientation of the muscle fibers, in accordance with the SENIAM project (Hermens et al., 1999). The inter-electrode distance was maintained at 30 mm, and a reference electrode was placed over the left proximal tibia (Chimera et al., 2004). Muscle activation data were obtained for three executions of each type of the jump, at a sampling frequency of 2000 Hz, in accordance with the Nyquist theorem (Hori et al., 2009). An electromyograph (model Miotool Wireless, MIOTEC Biomedical Equipment; Porto Alegre, Brazil) with an eight-channel system, which was fixed to the waist of each skater with a strap, was used. The signal was transmitted wirelessly to a laptop computer (DELL E5410, São Paulo, Brazil) and recorded using Miotec Studio software (version 1.2, MIOTEC Biomedical Equipment; Porto Alegre, Brazil). Before the tests the skaters performed a specific warm-up that consisted of stretching and single jumps with skates, in the same way that they are used to do in their practices. Two to four practice trials were allowed for each jump prior to the collection of data. During the tests, athletes performed three trials of each type of the jump, assigned in a random order, with at least one minute rest interval, and only the best jump was selected for further analysis. The jump selected corresponded to the one that would have the higher scores in a competition. The criterion for selecting this jump was to perform the jump as close as possible to the rule requirements, without losing balance. These decisions were made by a roller figure skating coach.

The performances were videotaped to align the movements and the EMG signal. A marker in the Miograph software and an LED, which was placed on the camera, were used for the alignment (McNitt-Gray et al., 2001). The alignment was also used for the subsequent cutting of the EMG signal.

Furthermore, the EMG values were normalized by the maximal voluntary isometric contraction (MVIC) of each of the various evaluated muscles. Subjects were instructed about the procedures and they were familiarized with the protocol. Each MVIC was performed within 5 s against resistance and it was repeated three times, with a 2 min rest interval. We provided verbal encouragement to motivate all subjects to achieve their maximal voluntary contraction levels. Subjects were positioned on exercise machines, fitted with a load cell coupled to the cable that displaced the load. The load cell was connected to an A/D converter (MIOTEC, Porto Alegre, Brazil) and was calibrated prior to the data collection, according to the manufacturers’ instructions. The load cell was used to allow the analysis of the EMG signal in the most stable phase of the force production. Hip, knee and ankle angles were measured with a goniometer (PROFISIOMED, Porto Alegre, Brazil).

EMG signal during MVIC for the knee extensor muscles rectus femoris, vastus lateralis and vastus medialis, was obtained while the subjects were seated on a knee extension exercise machine (Taurus, Porto Alegre, Brazil) with the hip flexed at 90° and the left knee flexed at 120° (180° represented the full extension). For the biceps femoris muscle (short head), EMG signal during MVIC was obtained while the subjects were in a prone position with the left knee flexed at 120°, and for the gluteus maximus, it was obtained while the subjects were in a supine position with the left hip flexed at 90° and the left knee fully extended. Finally, for the tibialis anterior and lateral gastrocnemius, EMG signal during MVIC was obtained while subjects were in a standing position, placed on the top of a bench, with the left foot forward and the ankle in a neutral position.

### Analysis

The EMG signals that were obtained during the skaters’ jumps were analyzed using the videotaped performances. The EMG signals were analyzed using SAD32 software and filtered using a fifth-order Butterworth band-pass filter with cut-off frequencies ranging between 20 and 500 Hz. These signals were cut, and the slices corresponded to the duration of each jump, which was observed in the kinematic analysis. An RMS envelope with a *Hamming* moving window was used for the EMG signal analysis, and each window represented 15% of the total duration of each jump. The EMG signals during the MVIC were filtered with a Butterworth low-pass filter at a cut-off frequency of 9 Hz. In order to determine the highest MVIC, signals were sliced into 1 s segments. Each segment corresponded to the most stable phase, as verified by the signal obtained from the load cell that was used during the MVIC. The root mean square value (RMS) was then calculated for each muscle. Test-retest reliability coefficients (ICC) values were previously obtained for this method and ranged from 0.6 to 0.8.

### Statistical Analysis

Due to the limited number of subjects in the present study, only descriptive analysis was performed in this study. EMG activity patterns were evaluated for the group of skaters as a whole and were compared between each type of the jump as well as between the different phases of the jumps.

## Results

**Figure 2:**
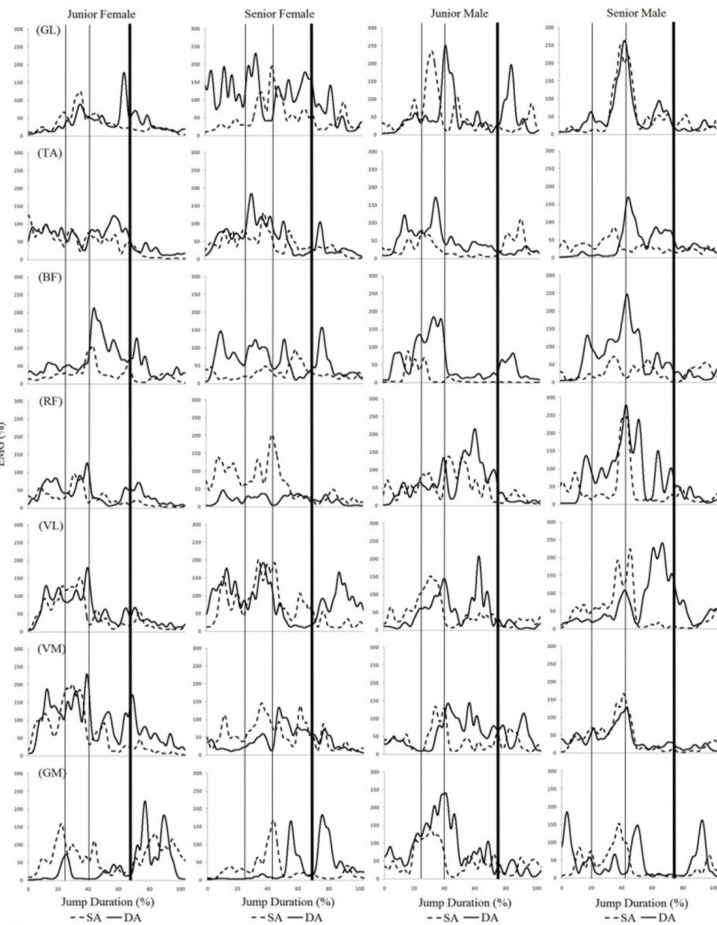
Electromyographic activity, normalized by MVIC, of the gastrocnemius lateralis (GL), tibialis anterior ITA), biceps femoris (BF), rectus femoris (RF), vastus lateralis (VL), vastus medialis (VM) and gluteus maximus (GM). The curves indicate the single Axel (SA) and double Axel (DA) with their four phases (preparation, take-off, flight and landing) performed by the four skaters. The space between the thin lines determines the take-off phase and the space between the second thin line and the thick line determines the flight phase of the jumps

**Figure 3:**
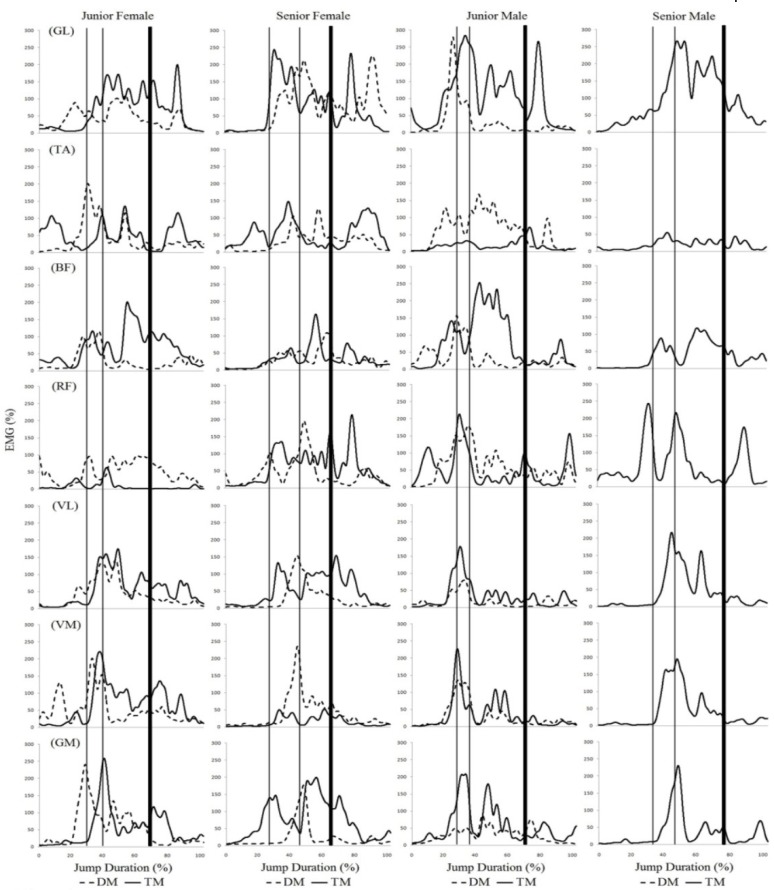
Electromyographic activity, normalized by MVIC, of the gastrocnemius lateralis (GL), tibialis anterior (TA), biceps femoris (BF), rectus femoris (RF), vastus lateralis (VL), vastus medialis (VM) and gluteus maximus (GM). The curves indicate the double Mapes (DM) and triple Mapes (TM) with their four phases (preparation, take-off, flight and landing) performed by the four skaters. The space between the thin lines determines the take-off phase and the space between the second thin line and the thick line determines the flight phase of the jumps

The results of the electromyographic activity of each jump are depicted in Figures 2 and 3. Due to a loss of data, the results from the senior male skater for the double Mapes jump are not presented.

Analysis of the muscle activity curves indicated that, in general, the *gastrocnemius lateralis, rectus femoris, biceps femoris* and *vastus lateralis* muscles presented greater activity and longer duration (that is, more phases) during the jumps with more rotations (double Axel and triple Mapes). There was also more activity in the *gluteus maximus* during the triple Mapes jump than during the double Mapes jump. The *gluteus maximus* also appeared to be more active during the triple Mapes than during the double Axel. The muscle activity during the different phases of the Axel and Mapes jumps was compared, and almost all muscles showed greater activity during the take-off and flight phases, especially for the jumps with more rotations (double Axel and triple Mapes). For these jumps, especially the triple Mapes, the *biceps femoris* appeared to be active most frequently during the flight phase.

Differences between male and female skaters were observed during the landing phase of the triple Mapes. It appeared that female skaters demonstrated higher muscle activities in TA, VL, VM and GM during this phase, when compared to their male counterparts.

## Discussion

The primary findings of the present study were that almost all of the evaluated muscles showed greater activity during the jumps with more rotations than during the jumps with fewer rotations. In addition, the muscles were frequently more active during the take-off and flight phases, especially in the jumps with more rotations. These results confirm the hypothesis of the present study. Some researchers who have analyzed ice figure skating argued that the activity of some muscle groups, such as the *quadriceps, hamstrings* and *gastrocnemius*, as well as the activity of the *gluteus maximus*, is critical to the success of figure skating jumps (Aleshinsky et al., 1988; Poe et al., 1994; King, 2005). The contribution of the muscles will also vary depending on the type of the jump performed (forexample, Toe Loop or Mapes, Axel, Flip), as observed in the present study for the *gluteus maximus* because this muscle was more active during the triple Mapes jump than during the double Axel jump. One reason for this pattern may be the different techniques employed in each jump. For instance, the take-off in the Mapes jump is executed from the toe pick, whereas the take-off in the Axel jump is executed from the forward outside edge of the skate.

Analysis of the neuromuscular pattern of the jumps that were evaluated in the present study indicated that the activity in the *biceps femoris, gastrocnemius lateralis, rectus femoris, vastus lateralis* and *gluteus maximus* muscles (particularly for the triple Mapes jump for the latter muscle) for jumps with the greatest number of rotations, mostly during the take-off and flight phases, was greater than that for the jumps with fewer rotations. These results are consistent with those obtained in the study by Taylor and Psycharakis (2009), who showed greater activation of the muscles of the take-off leg during jumps with more rotations. This study evaluated the *gastrocnemius medialis, rectus femoris, biceps femoris* and *adductors muscles* of a national-level ice skater performing single and double toe loop and flip jumps.

From the analysis of the various phases of the jumps, the contribution of the *gastrocnemius lateralis* appeared to be greater during the take-off phase of the jumps analyzed in the present study, which may indicate a greater use of plantar flexion during this phase. Moreover, the *biceps femoris* muscle appears to be frequently activated during the flight phase of the jumps with more rotations (especially during the triple Mapes jump). A greater contraction of this muscle may occur to maintain a closed position during the flight phase, during which participants compress the left leg against the right. This position may also explain the longer activation duration of some muscles during the jumps with more rotations. Furthermore, the greater BF activity during the flight phase of the triple Mapes might be a result of anti-inertial moments. The hip extensor moment might work to overcome the hip flexor inertia seen in faster rotations.

During the landing phase of the triple Mapes, the female skaters appear to activate more the TA, VL, VM and GM muscles, when compared to their male counterparts. It is interesting to note that these are all uniarticular muscles and that the differences of the biarticular muscles RF and GL, do not appear to be as strong. These muscles seem to demonstrate greater activity during the landing of both male and female skaters, indicating their importance during this phase. Perhaps the greater activity observed only for the female skaters for the uniarticular muscles reflects more difficulty while finalizing the jump, and a greater effort that these skaters probably had to make to better control the left leg during landing. During the beginning of the landing phase, the skaters are controlling the left leg, so that the left skate does not touch the ground. After, they move the left or free leg, backwards, to end the jump properly. Analyzing the video recordings, it can be observed that the females evaluated in the present study control the left leg with much more effort and tension, since they land with the left skate closer to the ground. The TA is probably active to control the left skate, eccentrically, so that it does not touch the ground and the VL and VM muscles are probably active to maintain the knee extended, helping to control the movement of the left leg. The GM is probably active because of the hip extension during the jump’s finalization. In contrast, the males appear to perform the landing with less effort, having a more natural movement of the left leg during landing, i.e. the left skate is not that close to the ground, and they are more ready to end the jump moving the left leg backwards. Therefore, the male skaters in the present study did not need to activate the TA, VL, VM and GM to better control the left leg during landing. This could be one reason for the greater muscle activity observed during the landing phase for the females in the present study. However, more studies evaluating the control of the free leg during the landing phase of roller figure skating jumps are required to better understand this process.

The information about the muscle activity during different figure skating jumps may be important for guiding the specific preparation of these athletes, with the appropriate selection of the exercises. Plyometric training is an example of specific preparation that can be used by skaters and stimulates the neuromuscular system, promoting improvement in jump performances (Ebben and Watts, 1998; Santos and Janeira, 2008; Lephart et al., 2005). Wu et al. (2010) analyzed the vertical jump (countermovement jump) height and the activity of the *gastrocnemius lateralis* and *soleus* muscles before and after eight weeks of plyometric training. The researchers found an increase in both vertical jump height and muscle activity, although the increase in the activity was only statistically significant for the *soleus*. In the present study, the technical elements of figure skating were not evaluated after a specific type of training. Nevertheless the information regarding the main muscles that were activated during roller figure skating jumps may be of great value for prescribing plyometric training. Furthermore, it is important for future studies to examine the effects of specific training programs, such as plyometric training, on roller figure skaters. Forinstance, the result of the present study, in which an increase in muscular activity was observed during the landing phase of the triple Mapes, specially for the female skaters, suggests that higher volumes of exercises which develop the leg muscles eccentrically for landing, such as box landings and balancing on a wobble board while imitating the landing motion of the free leg (left leg for the Axel and Mapes jump) (KING, 2005), will probably be of great importance during athletes’ transitioning to include the triple Mapes in competitions. The female skaters evaluated in the present study will probably benefit from practicing these exercises, which will help them to perform the triple Mapes landing effortlessly, regarding the movement of the free leg.

In conclusion, there was more activity in the *biceps femoris, gastrocnemius lateralis, rectus femoris, vastus lateralis* and the *gluteus maximus* muscles during jumps with a larger number of rotations for the athletes in this study, especially during the take-off and flight phases. Furthermore, the activity of the *gluteus maximus* muscle appeared to be greater during the triple Mapes than during the double Axel, and the *biceps femoris* demonstrated more activation during the flight phase of the jumps with more rotations, especially during the triple Mapes jump. The results in the present study cannot be generalized because only a small number of elite skaters were evaluated; however, these results may provide a useful contribution to understanding the muscle activity pattern during figure skating jumps at the highest level of competitive performance.

For practical applications, these results are important in identifying which muscles are more involved in roller figure skating jumps. The results are also helpful for planning specific training programs for these muscles, which may provide benefits to elite athletes and, most likely, to skaters of other levels who aspire to perform triple jumps and succeed at international level. Plyometric exercises performed in a manner that closely resemble the movements used in figure skating (for instance, unilateral) may be used. Thus, these exercises might emphasize concentric knee extension and plantar flexion during take-off, with the goal of increasing the activity of the *quadriceps* and *gastrocnemius* muscles. To increase the activity of the evaluated muscles during jumps with more rotations, exercises that simulate the flight phase may be performed. During the flight phase, the athlete holds the left leg against the right, activating the BF muscle as a result of anti-inertial moments. This situation may be simulated by applying resistance to the left leg, making possible for the skater to work on overcoming the hip flexor inertia, seen with faster rotations.

Moreover, the specific training programs may mainly exercise muscles that are essential for performing the jumps in the skater’s routines. For instance, exercises that simulate the movements of the left leg during the triple Mapes jump, in which the skater jumps from the toe pick of the skate, may be imperative for increasing the *gluteus maximus* activity in a specific manner.

Finally, there have been no studies investigating neuromuscular activity during roller figure skating. Roller figure skating is becoming more popular, and information regarding the muscle activation during different types of figure skating jumps may be important for coaches. In addition, figure skating on ice and roller figure skating may be similar in many technical and biomechanical aspects. We cannot find studies in which these parameters have been measured in roller figure skaters, and those measurements are necessary to compare the two types of skating. This paper is the first attempt to provide information concerning the movements practiced by roller figure elite skaters and provides further insight regarding differences and similarities between roller and ice figure skating. Unfortunately, it was not possible to evaluate skaters of different levels, since the analysis included triple jumps that only elite skaters are able to perform. We suggest that further studies evaluate the neuromuscular response of roller figure skating jumps that are made by both elite and intermediate level skaters.
